# Low-Load Conditioning with Blood Flow Restriction and Whole-Body Vibration Induces Post-Activation Performance Enhancement Effects and Improves Anaerobic Performance: A Pilot Study

**DOI:** 10.5114/jhk/202048

**Published:** 2025-11-20

**Authors:** Ruibin Guo, Yinglu Hong, Nan Xu, Hongwen Wei

**Affiliations:** 1 School of Strength and Conditioning Training, Beijing Sport University, Beijing, China.; 2 Movement and Cognitive Rehabilitation Science Program, Department of Kinesiology and Health Education, University of Texas at Austin, Austin, the United States of America.; 3 Department of Health Promotion and Rehabilitation, Lithuanian Sport University, Kaunas, Lithuania.

**Keywords:** Wingate test, blood lactate, warm-up, activation exercise

## Abstract

This study examined the post-activation performance enhancement (PAPE) effects of low-load conditioning (30% of 1 repetition maximum (RM) squat) with blood flow restriction (BFR) or whole-body vibration (WBV) only, and with both BFR and WBV on anaerobic performance. Forty anaerobically-trained men were randomly allocated into one of the four groups: CON, BFR, WBV or BFR+WBV, and completed five visits: Visit 1: 1RM test and familiarization session; Visit 2: Wingate test; and Visits 3 to 5: conditioning according to the group assignment, followed by a Wingate test after different rest intervals: 4 (T4), 8 (T8) or 12 (T12) min on each visit, respectively, in randomized order. Capillary blood lactate samples were collected at the 3^rd^, the 5^th^, the 8^th^ and the 10^th^ min after the Wingate test. Analysis revealed significant interaction effects between group and time on peak blood lactate concentration (p < 0.019). The within-group analysis showed that compared to PRE, 1) mean power (mean-P), mean power/body mass (mean-P/BM) and total work (TW) of all three groups were significantly greater at T8 (p < 0.045); 2) peak power and peak power/body mass of the BFR and the WBV group were greater at T8 (p < 0.024); 3) minimal power of the BFR+WBV group was significantly lower, and fatigue slopes were significantly greater at T8 (p < 0.032); and 4) mean-P, mean-P/BM and TW of the BFR+WBV group were significantly greater at T4 (p < 0.015). Low-load conditioning combined with BFR or WBV may induce potential PAPE to enhance anaerobic performance, and the combined-type of the protocol may induce such benefits in a faster time fashiont.

## Introduction

Incorporating an appropriate warm-up protocol is crucial for athletic performance and can help reduce the risk of injury for athletes during competition or training. Post-activation performance enhancement (PAPE), which can be induced by warm-up protocols, has garnered increasing research attention. PAPE may enhance voluntary dynamic force production following a prior bout of a conditioning activity (CA), thereby benefiting subsequent athletic performance (Blazevich and Babault, 2019).

Different CA protocols have been utilized to induce PAPE, with high-load CAs such as squats at 75–95% of 1 repetition maximum (1RM) being the most commonly employed (Wilson et al., 2013). For instance, Cuenca-Fernández et al. (2017) observed PAPE in squat jumps among trained swimmers following various CAs (back squat, bench press or a combination of both) involving four repetitions at 90% 1RM. However, it is important to consider several drawbacks associated with these high-load training protocols. High-load training can lead to significant fatigue, which may limit subsequent potentiation and consequently reduce athletic performance (MacIntosh et al., 2008).

To address the issues associated with high-load training protocols, recent research has focused on exploring alternative training methods that may induce PAPE, such as isometric contractions (Zero and Rice, 2021), eccentric contractions (Stastny et al., 2024), plyometric jumps (Tobin and Delahunt, 2014), and electromyostimulation (Sari et al., 2022). Among these, low-load training (e.g., less than 50% of 1RM (Lixandro et al., 2018)) combined with blood flow restriction (BFR) or whole-body vibration (WBV) has been suggested as a promising strategy to induce PAPE and related benefits (Chen et al., 2017). For example, studies have shown that BFR, which partially restricts arterial inflow and fully restricts venous outflow in working muscles during exercise (Scott et al., 2014), when combined with low-intensity resistance training, can achieve effects on athletic performance comparable to those of traditional high-load training (Doma et al., 2020). Similarly, WBV, a method that facilitates reflexive activation of alpha motor neurons, increases spatial recruitment (Wuestefeld et al., 2020), and improves power and strength (Peng et al., 2025), combined with low-load training, may help enhance the recruitment of higher threshold motor units (Sweeney et al., 1993), leading to similar but more sustained improvements in muscular power compared to heavy-load resistance training (Cochrane et al., 2010). Additionally, a study by Centner et al. (2019) indicated that implementing BFR together with WBV could acutely enhance improvements beyond those achieved by WBV alone, as evidenced by higher lactate concentrations and increased EMG amplitude in lower limb muscles. Due to the combination of metabolic and neural factors, in BFR, reduced muscle oxygen availability and increased blood lactate concentration result in recruitment of more muscle fibers, while in WBV, the vibration leads to more neuromuscular stimulation. Based on the phenomenon basis and main mechanism of BFR + WBV observed in EMG, whether performance can be “1 + 1 ≥ 2” is interesting. The idea that the phenomenon returns to the mechanism, and that the “combined mechanism” then acts on the phenomenon should be put into practice. However, the effects of these two novel training protocols, as well as their combined use, on facilitating PAPE during anaerobic cycling tests have not been explicitly examined and compared. Doing so would provide critical knowledge for the appropriate design of protocols to facilitate PAPE in future research and training practices, helping maximize the benefits related to PAPE for athletic performance.

Therefore, this pilot study aimed to investigate the effects of low-load conditioning combined with BFR and WBV on PAPE during anaerobic exercise in younger male adults. The hypothesis was that all three conditioning protocols would significantly enhance anaerobic capacity by promoting PAPE, with the combined BFR and WBV protocol expected to yield the greatest improvement compared to BFR or WBV alone.

## Methods

### Participants

Forty-eight participants were recruited from the Beijing Sport University. The inclusion criteria required participants to be between 18 and 35 years old and anaerobically trained, meaning they engaged in resistance training, Olympic lifting, sports involving repeated anaerobic exercises such as sprinting and jumping, or anaerobic training at least twice a week for six months prior to the study (Doma et al., 2020). Exclusion criteria were acute illness, injury, unstable medical conditions, hospitalization in the past three months, musculoskeletal diseases, pain, orthopedic problems affecting exercise performance, and use of certain medications.

### Ethics

Participants were fully informed of the study’s benefits and risks, and informed that several blood samples would be collected for blood lactate concentration testing. Afterwards, they provided signed consent following the Helsinki Declaration guidelines. The study was approved by the Sports Science Experiment Ethics Committee of the Beijing Sport University, Beijing, China (protocol code: 2020079; approval date: 27 April 2020). No minors participated in the study.

### Experimental Design

Participants were randomly assigned to one of the four groups: squat conditioning only (CON group), squat conditioning with BFR (BFR group), squat conditioning with WBV (WBV group) or squat conditioning with both BFR and WBV (BFR+WBV group). Each participant completed five study visits with a one-week rest period in between. Prior to each visit, participants were instructed to avoid exercise that could cause fatigue for 48 hours prior to exercise, as well as alcohol, caffeine, and other supplements for 24 h. The first visit included a familiarization session with a standardized warm-up protocol, training specific to their group, a Wingate test, and a 1RM test. The warm-up consisted of leg swings and cycling on an ergometer at 60 W for five minutes, with a maximal effort for 10 s. On the second visit, participants completed the Wingate test 2 min after the warm-up. During Visits 3 to 5, participants performed conditioning according to their group assignment, followed by a Wingate test after different rest intervals (i.e., 4 (T4), 8 (T8) or 12 (T12) minutes) on each visit, respectively, in randomized order (Lowery et al., 2012). Group allocation and testing order were randomized using a random number generator in R, ensuring that each participant experienced different intervals between conditioning and testing protocols in randomized order.

**Figure 1:**
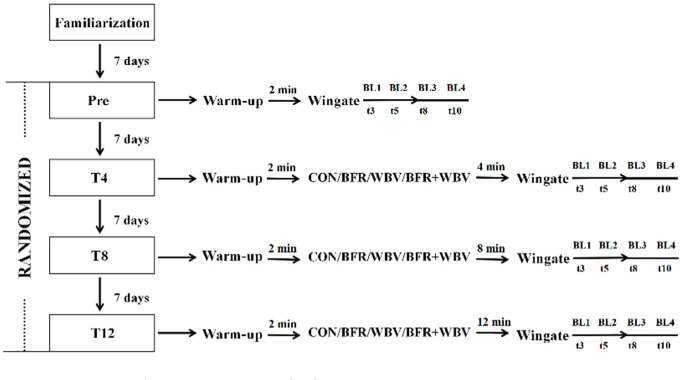
Research design overview.

Capillary blood lactate samples were collected at the 3^rd^, the 5^th^, the 8^th^ and the 10^th^ min after each Wingate test during Visits 2 to 5 (Simões et al., 2016). The blood samples were analyzed using the YSI 2300 Stat Plus and stored in EDTA tubes with sodium fluoride to prevent coagulation. Subsequently, they were transported to the laboratory for analysis. Further analysis of the blood lactate samples was conducted using the EKF Biosen C-Line. The peak blood lactate value was determined as the maximum value from the measurements taken at the 3^rd^, the 5^th^, the 8^th^, and the 10^th^ min after each Wingate test. The research design is presented in Figure 1.

### Conditioning Protocols

The conditioning protocols for all four groups consisted of three sets of 15 squats at 30% of each participant’s 1RM, performed with 90- degree knee flexion. Each repetition included a 2-s eccentric phase and a maximum speed concentric phase (Wilk et al., 2020). A digital metronome ensured consistent timing across all conditions, with a 30-s rest interval between each set (Luebbers et al., 2019). Participants in the CON group performed squat conditioning only. In the BFR group, after warming up, participants wore pressure cuffs of 9-cm width (B-Strong^TM^, USA) on the most proximal part of each leg, inflated to 140 mmHg, which remained inflated during the squats. The pressure was released immediately after the squats, and the Wingate test was performed without BFR. This pressure level effectively restricts arterial flow and occludes venous return, as suggested by previous research (Kojima et al., 2021). In the WBV group, participants performed dynamic squats on a vibration platform (Pro5 Vibration Plate, Power Plate, United States of America) wearing non-slip shoes, holding a barbell, and maintaining a neutral head position to minimize vibration transmission. The platform operated at vertical 40 Hz for 30 s, a frequency shown to elicit the highest reflex response (Ritzmann et al., 2013), with amplitude ramping up from 2 to 4 mm. After WBV, researchers enquired for side effects like muscle trembling, tingling or weakness in the knees. In the BFR+WBV group, participants followed the WBV protocol with the addition of the same blood flow restriction protocol as the BFR group.

### Wingate Test

The 30-s Wingate test was performed on a cycle ergometer with resistance set at 7.5% of each participant’s body mass. This test is widely recognized for its validity and reliability in assessing anaerobic capacity (Bar-Or, 1987; Maciejewski et al., 2016). Performance was evaluated using Monark Anaerobic test software, focusing on primary outcomes: peak power (Peak-P; the power output in the first 5 s), mean power (Mean-P; the average power output over 30 s), minimum power (Min-P; the lowest power generated during the test), and the fatigue slope (FTGSlope; the rate of power decline). Secondary outcomes included a peak power to body mass ratio (Peak-P/BM), a mean power to body mass ratio (Mean-P/BM), and total work (TW).

### Statistical Analysis

Statistical analyses were performed using SPSS 26.0 (IBM, Chicago, IL, USA) with a significance level set at *p* < 0.05. Demographic characteristics and study outcomes were summarized using descriptive statistics (mean and standard deviation). Two-way repeated-measures ANOVA models were utilized to compare the impact of various conditioning protocols on PAPE facilitation among groups. The Shapiro-Wilk test was used to assess the normality of the data distributions. The Mauchly’s test of sphericity was conducted to evaluate the sphericity assumption for time points. If the sphericity assumption was violated, the Greenhouse-Geisser correction was applied to adjust the degrees of freedom. The homogeneity of variances across groups was examined using the Levene’s test. The dependent variables included outcomes from the Wingate test (Peak-P, Mean-P, Min-P, FTGSlope, Peak-P/BM, Mean-P/BM, and TW) as well as peak blood lactate. The model factors consisted of group (CON, BFR, WBV, and BFR+WBV) and time points (PRE, T4, T8, and T12) along with their interactions. Significant interactions were further analyzed using Bonferroni's post-hoc tests to identify specific differences. When significant interactions were not present, one-way repeated-measures ANOVA was conducted to assess the effects of conditioning protocols within each group from baseline (PRE) to follow-up (T4, T8, and T12). Effect sizes were evaluated using Cohen’s *d*, with values categorized as small (< 0.5), moderate (0.5 to 0.8), and large (> 0.8) (Cohen, 1988).

## Results

Forty-eight participants were initially recruited, but three were excluded due to noncompliance and five did not meet the inclusion criteria. Thus, 40 participants (n = 10 per group) completed the study, and their data were successfully recorded. The demographics and baseline characteristics are detailed in Table 1. There were no significant differences in Wingate test outcomes or peak blood lactate concentration at baseline between groups (*p* > 0.282).

**Table 1:** The demographics and the baseline characteristics of the participants.

Group	N	Age (yrs)	Body height (cm)	Body mass (kg)	Squat-1RM (kg)
All	40	21.7 ± 1.2	179.2 ± 4.6	75.0 ± 4.6	145.3 ± 9.3
CON	10	22.0 ± 1.6	178.3 ± 4.4	74.9 ± 2.3	147.5 ± 8.0
BFR	10	22.0 ± 1.8	179.7 ± 4.3	77.2 ± 5.0	147.0 ± 7.0
WBV	10	21.0 ± 1.0	179.4 ± 4.5	75.7 ± 4.1	148.5 ± 6.5
BFR+WBV	10	21.6 ± 1.3	177.7 ± 4.4	74.4 ± 7.3	149.6 ± 8.7

CON = control group; BFR = BFR group (squat conditioning with blood flow restriction); WBV = WBV group (squat conditioning with whole-body vibration); BFR+WBV = BFR+WBV group (squat conditioning with both blood flow restriction and whole-body vibration

### Effects of Conditioning Protocols on PAPE in Anaerobic Performance

The primary two-way repeated-measures ANOVA showed significant main effects of time on Mean-P (*p* < 0.001), FTGSlope (*p* < 0.03), Mean-P/BM (*p* < 0.001), and TW (*p* < 0.001), but no significant interaction between time and group or main effects of group (*p* > 0.714). Post-hoc analyses indicated that Mean-P, Mean-P/BM, and TW were significantly greater at T4 (*p* < 0.005) and T8 (*p* < 0.003) compared to PRE, with no significant changes observed for FTGSlope (*p* > 0.061), and no significant effects on Peak-P and Min-P (*p* > 0.152). Secondary one-way repeated-measures ANOVA revealed that compared to PRE, both Mean-P and Mean-P/BM were significantly greater at T8 in the BFR (*p* < 0.005, ES = 0.38–0.62), WBV (*p* < 0.012, ES = 0.33–0.59), and BFR+WBV (*p* < 0.045, ES = 0.33–0.78) groups. Both Peak-P and Peak-P/BM were significantly greater at T8 in the BFR (*p* < 0.001, ES = 0.47–0.58) and WBV (*p* < 0.024, ES = 0.27–0.78) groups. Min-P was significantly lower (*p* < 0.012, ES = 1.11) and FTGSlope significantly higher (*p* < 0.032, ES = 1.09) at T8 in the BFR+WBV group. TW was significantly greater at T8 in the BFR (*p* < 0.005, ES = 0.62), WBV (*p* < 0.011, ES = 0.59), and BFR+WBV (*p* < 0.044, ES = 0.78) groups compared to PRE. Uniquely, Mean-P (*p* < 0.015, ES = 1.11), Mean-P/BM (*p* < 0.014, ES = 0.32), and TW (*p* < 0.014, ES = 0.77) were significantly greater at T4 in the BFR+WBV group. For detailed results, see Table 2a and 2b.

**Table 2a:** Anaerobic performance for a 30-s Wingate test and PBL following BFR, WBV, BFR+WBV at T4, T8 and T12 (mean ± SD).

	CON				BFR				
	PRE	T4	T8	T12	PRE	T4	T8	T12
**Peak-P**	960.00 ± 43.86	969.30 ± 58.70	959.59 ± 55.36	973.40 ± 48.40	958.70 ± 44.65	967.80 ± 43.84	985.90 ± 49.00*	970.80 ± 50.74
**Peak** **-P/BM**	12.83 ± 0.59	12.96 ± 0.86	12.84 ± 0.89	13.04 ± 0.99	12.71 ± 0.88	12.83 ± 0.82	13.07 ± 0.92*	12.87 ± 0.96
**Mean-P**	610.30 ± 27.14	613.90 ± 29.04	616.40 ± 28.68	615.20 ± 31.59	611.10 ± 30.27	624.80 ± 36.37	631.60 ± 35.80*	616.90 ± 28.66
**Mean-P/BM**	8.17 ± 0.16	8.22 ± 0.18	8.25 ± 0.15	8.24 ± 0.20	8.12 ± 0.25	8.30 ± 0.28	8.38 ± 0.25*	8.19 ± 0.24
**Min-P**	422.70 ± 43.66	422.90 ± 30.36	394.80 ± 40.85	430.80 ± 26.93	421.00 ± 56.31	401.70 ± 47.06	421.90 ± 56.26	408.20 ± 49.57
**FTG** **Slope**	55.95 ± 4.36	56.16 ± 7.74	58.87 ± 3.21	55.73 ± 1.89	56.19 ± 4.47	58.54 ± 4.00	57.20 ± 5.27	57.98 ± 3.99
**TW**	18019.40 ± 252.33	18120.80 ± 270.07	18198.50 ± 268.87	18165.50 ± 295.41	18041.10 ± 281.32	18449.20 ± 339.90	18644. 90 ± 335.06*	18210.60 ± 268.75
**PBL**	15.59 ± 1.19	15.97 ± 1.23	15.94 ± 1.41	15.46 ± 1.65	15.60 ± 1.46	17.82 ± 1.52	18.27 ± 1.35*^#^	17.40 ± 1.29

* Significantly different from PRE, p < 0.05; # Significantly different from CON, p < 0.05; Peak-P = Peak power; Peak-P/BM = Peak power per body mass; Mean-P = Mean power; Mean-P/BM = Mean power per body mass; Min-P = Minimal power; FTG Slope = Fatigue Slope; TW = Total work; PBL = Peak blood lactate; CON = control group; BFR = BFR group (squat conditioning with blood flow restriction); WBV = WBV group (squat conditioning with whole-body vibration); BFR+WBV = BFR+WBV group (squat conditioning with both blood flow restriction and whole-body vibration)

**Table 2b:** Anaerobic performance for a 30-s Wingate test and PBL following BFR, WBV, BFR+WBV at T4, T8 and T12 (mean ± SD).

	WBV				BFR+WBV			
	PRE	T4	T8	T12	PRE	T4	T8	T12
**Peak-P**	958.30 ± 37.07	956.90 ± 54.57	984.30 ± 28.91*	959.90 ± 52.35	963.50 ± 61.45	972.90 ± 61.68	974.80 ± 49.54	959.00 ± 57.35
**Peak** **-P/BM**	12.50 ± 1.20	12.48 ± 1.29	12.82 ± 1.03*	12.52 ± 1.25	13.14 ± 1.97	13.25 ± 1.84	13.27 ± 1.79	13.05 ± 1.70
**Mean-P**	612.20 ± 39.33	623.90 ± 37.71	633.90 ± 34.13*	620.70 ± 31.63	608.70 ± 21.84	632.50 ± 37.90*	631.80 ± 35.97*	621.40 ± 34.72
**Mean-P/BM**	7.96 ± 0.20	8.12 ± 0.20	8.25 ± 0.20*	8.08 ± 0.20	8.27 ± 0.28	8.59 ± 0.31*	8.57 ± 0.30*	8.44 ± 0.31
**Min-P**	421.80 ± 53.70	404.60 ± 34.73	408.90 ± 21.25	407.90 ± 36.81	432.70 ± 24.35	404.60 ± 54.72	400.80 ± 32.47*	401.00 ± 61.36
**FTG** **Slope**	56.02 ± 4.76	57.75 ± 2.36	58.41 ± 2.89	57.49 ± 3.35	55.00 ± 2.70	58.43 ± 4.93	58.77 ± 4.06*	58.17 ± 6.00
**TW**	18068.30 ± 367.71	18414.80± 350.72	18707.50± 318.31*	18319.40± 294.01	18025.30± 204.07	18735.40± 355.61*	18710.20± 335.74*	18402.40± 323.76
**PBL**	15.83 ± 1.65	17.62 ± 1.50	18.53 ± 1.43*^#^	17.33 ± 1.26*^#^	15.84 ± 1.06	18.58 ± 1.13*^#^	18.64 ± 1.55*^#^	17.02 ± 0.90

* Significantly different from PRE, p < 0.05; # Significantly different from CON, p < 0.05; Peak-P = Peak power; Peak-P/BM = Peak power per body mass; Mean-P = Mean power; Mean-P/BM = Mean power per body mass; Min-P = Minimal power; FTG Slope = Fatigue Slope; TW = Total work; PBL = Peak blood lactate; CON = control group; BFR = BFR group (squat conditioning with blood flow restriction); WBV= WBV group (squat conditioning with whole-body vibration); BFR+WBV = BFR+WBV group (squat conditioning with both blood flow restriction and whole-body vibration)

### Effects of Conditioning Protocols on Peak Blood Lactate

The two-way repeated-measures ANOVA showed significant main effects of group (*p* < 0.001), time (*p* < 0.001), and their interaction (*p* < 0.019) on peak blood lactate. Post-hoc analyses revealed that peak blood lactate in the BFR, WBV, and BFR+WBV groups was significantly higher than in the CON group (*p* < 0.007). Compared to PRE, peak blood lactate was higher at all post-intervention times (T4, T8, T12) (*p* < 0.004), with the highest levels at T8. Significant increases in peak blood lactate at T8 were observed in the BFR (ES = 1.61–1.87), WBV (ES = 1.50–2.06), and BFR+WBV (ES = 1.57–2.13) groups, and at T12 in the WBV group (ES = 0.4–1.52). Additionally, the BFR+WBV group showed significantly higher peak blood lactate at T4 compared to PRE and to T4 and T8 in the CON group (ES = 0.91–1.17). For detailed results, see Table 2.

## Discussion

This pilot study is the first to investigate and compare the effects of low-load conditioning with BFR or WBV alone, as well as with both BFR and WBV, on enhancing PAPE during anaerobic capacity testing. No significant differences were found between the conditioning protocols, however, within-group analysis indicated that the novel conditioning protocols using BFR and WBV may enhance PAPE, leading to significant improvements in anaerobic performance over time within the group. Additionally, it was suggested that the combined protocol could induce PAPE faster, approximately four minutes earlier. In addition to traditional high-load protocols, PAPE can also be achieved through compound conditioning. In this study, BFR and WBV protocols were used to stimulate a certain amount of muscle to cause the “balance” state of fatigue and activation. Seizing the advantage opportunity of activation can enhance performance. The potential benefits of combining BFR and WBV with low-intensity squats to induce PAPE on anaerobic performance may be attributed to increased muscular responses, resulting in higher power output from the lower limbs, as well as the sustained high power endurance stimulated by BFR and WBV (Giltay et al., 2014; Wernbom et al., 2009). BFR-induced metabolic accumulation (e.g., lactic acid) leads to cell swelling (Patterson et al., 2013), higher order motor unit recruitment (Fatela et al., 2019), and possibly a smaller muscle fiber pennation angle (Martín-Hernández et al., 2013), which enhances muscle fiber force transmission during physical activity (Folland and Williams, 2007). Preconditioning with WBV can increase twitch potentiation, the rate of force development, and power output in subsequent tasks (Cochrane et al., 2010). Appropriate WBV can enhance brain excitability (Tan et al., 2024), which refers to the ability of brain cells to generate electrical impulses in response to stimuli, influencing various functions such as muscle contraction and strength, which may be potential mechanisms of PAPE. Besides these “muscle-memory mechanisms” (Blazevich and Babault, 2019), such effects may also stem from the warm-up effect, including muscle temperature changes and intramuscular fluid accumulation (Blazevich and Babault, 2019). BFR can induce maximal artery dilation (Agewall et al., 1999), increase reactive hyperemia (Yanagisawa and Fukutani, 2018), and elevate muscle oxygenation post-BFR (Yanagisawa and Fukutani, 2018). These BFR-induced acute endothelial and metabolic adaptations are similar to those of a warm-up protocol, thus enhancing subsequent performance (Fortin and Billaut, 2019). Similarly, WBV can boost oxygen uptake (Cochrane et al., 2008) and muscle temperature (Cochrane et al., 2008) more effectively than traditional warm-up methods like stationary cycling or a passive warm-up. However, the exact mechanisms through which WBV and BFR induce these functional improvements remain unclear. Future studies should focus on elucidating these bio-neurophysiological elements to optimize BFR and WBV protocol designs for maximizing PAPE benefits.

The lack of significant differences between protocols may be due to the small sample size in this pilot study. Additionally, the mismatch between the squat as a conditioning activity and cycling as the subsequent test might have contributed to the lack of significance. According to the principle of dynamic correspondence, training protocols that mimic the task’s motion may provide greater benefits (Suarez et al., 2019). Studies have shown that conditioning activities resembling the subsequent task (e.g., cycling) can enhance performance more effectively than dissimilar activities (Lee et al., 2024; Skof and Strojnik, 2006). Therefore, it is essential to confirm the PAPE effect and refine these protocols considering the task’s specific requirements to maximize their benefits for PAPE and improve anaerobic performance in follow-up exercises or competitions.

Within-group analysis showed that the combined protocol could improve anaerobic performance at least four minutes earlier than the other two protocols, with sustained improvements for at least four minutes. This quicker improvement induced by BFR+WBV may be supported by the larger effect size of the increase in blood lactate concentration in the BFR+WBV group compared to the other groups. While not fully understood, blood lactate concentration is a crucial indicator of metabolic accumulation, reflecting increased type-II muscle fiber recruitment and greater anaerobic metabolism, both vital for anaerobic capacity improvement (Lüscher et al., 1983). Studies have linked performance enhancement to higher blood lactate concentration, suggesting that higher lactate accumulation correlates with greater enhancement (García-Pinillos et al., 2015; Skof and Strojnik, 2006). Overall, the findings indicate that both BFR and WBV hold promise for enhancing anaerobic performance. However, further research is needed to fully understand the underlying mechanisms and optimal variables of this combined protocol to maximize its effectiveness in athletic training and performance enhancement.

## Study Limitations and Future Recommendations

Several limitations of this study should be noted. This study focused on characterizing the effects of these novel conditioning protocols without including traditional high-load conditioning protocols for comparison. Additionally, our cohort was limited to younger, anaerobically trained men with a relatively small sample size, which may limit the generalizability of our findings to broader populations. Future studies should compare these novel protocols to traditional high-load conditioning and confirm the findings in larger, more diverse cohorts, including women and older athletes.

Despite using a randomized controlled design, order effects cannot be ruled out and should be examined in future studies. The BFR pressure in our study was set at a fixed level, which may not provide a consistent stimulus across participants due to individual physiological differences (e.g., vascular elasticity, muscle size, and shape). Future studies should customize the restriction pressure for each participant based on their limb occlusion pressure.

Physiological status, metabolic level, and other factors may also affect peak blood lactate concentration after the Wingate test. Thus, future studies should assess baseline blood lactate concentration and include it in the analysis and help better understand the rate of recovery from each PAPE condition. Finally, since the warm-up effect plays an important role in inducing PAPE, participants should complete a more comprehensive and task-specific warm-up to better assess the practical effects of conditioning activities in exercise or sports contexts, thereby enhancing external validity.

## Conclusions

This pilot study demonstrated that low-load conditioning combined with BFR, WBV, or both may induce a potential PAPE effect, enhancing anaerobic performance as early as four minutes post-conditioning. The combined protocol may yield these benefits more quickly, warranting further confirmation in future research.
